# Alarmingly high prevalence of hypertension and pre-hypertension in North India-results from a large cross-sectional STEPS survey

**DOI:** 10.1371/journal.pone.0188619

**Published:** 2017-12-21

**Authors:** Jaya Prasad Tripathy, Jarnail Singh Thakur, Gursimer Jeet, Sohan Chawla, Sanjay Jain

**Affiliations:** 1 International Union Against Tuberculosis and Lung Disease, The Union South East Asia Office, New Delhi, India; 2 Department of Community Medicine, School of Public Health, Post Graduate Institute of Medical Education and Research, Chandigarh, India; 3 Department of Internal Medicine, Post Graduate Institute of Medical Education and Research, Chandigarh, India; Shanghai Institute of Hypertension, CHINA

## Abstract

**Objectives:**

The study was primarily aimed at estimating the prevalence of hypertension and pre-hypertension and the risk factors of hypertension in the North Indian state of Punjab. It also aimed at assessing the magnitude of undiagnosed cases of hypertension in the community and ascertaining the blood pressure control status of those on treatment.

**Methods:**

A non-communicable disease risk factor survey (based on WHO-STEPS approach) was done in the state of Punjab, India in a multistage stratified sample of 5127 individuals. The study subjects were administered the WHO STEPS-questionnaire and also underwent anthropometric and blood pressure measurements.

**Results:**

Overall prevalence of HTN among the study participants was found out to be 40.1% (95% CI: 38.8–41.5%) whereas prevalence of pre-hypertension, isolated diastolic and isolated systolic hypertension were 40.8% (39.5–42.2%), 9.2% (8.4–10.0%) and 6.5% (5.9–7.2%) respectively. Age group (45–69 years), male gender, social group, marital status, alcohol use, obesity and salt intake (> = 5 gms/day) were the risk factors significantly associated with HTN. Among all persons with HTN, only 30.1% were known case of HTN or on treatment, among whom nearly 61% had controlled blood pressure. Patients with uncontrolled BP were more frequently male, obese patients, with sedentary lifestyle and patients with diabetes.

**Conclusions:**

The study reported alarmingly high prevalence of hypertension, especially of undiagnosed or untreated cases amongst the adult population, a significant proportion of whom have uncontrolled blood pressure levels. This indicates the need for systematic screening and awareness program to identify the undiagnosed cases in the community and offer early treatment and regular follow up.

## Introduction

In 2012, NCDs were responsible for around 38 million deaths per year, accounting for 68% of all deaths worldwide and 52% of all premature deaths. Over three quarters of those premature deaths were caused by cardiovascular diseases. CVD is the leading NCD which claimed 17.5 million lives in 2012 (46% of all NCD deaths).[1] Raised blood pressure (BP) (otherwise referred to as Hypertension or HTN) is the third most important attributable risk factor for burden of disease in South Asia (2010).[2] HTN is directly responsible for 57% of all stroke deaths and 24% of all coronary heart disease (CHD) deaths in India.[3]

Previous studies in India in the last decade have reported varying prevalence of hypertension ranging from 17–47% in the adult population. However, they have mostly been limited to specific population sub-groups and in certain geographical pockets.[4–12] A recent systematic review by Anchala et al. found the overall prevalence of hypertension in India to be 29.8% with significant urban-rural difference.[13] A large nationwide study (ICMR-INDIAB study) by Bhansali et al. reported hypertension among 26.3% of the population.[14] However, it covered only the Union Territory of Chandigarh from North India which does not truly represent the population. Because hypertension exerts a substantial impact on the cardiovascular health of the general population and enormous burden on the healthcare systems in India,[15] an estimation of its prevalence and identification of high risk groups is essential for planning of community based cardiovascular risk factor reduction interventions. According to previous studies, nearly 4/5^th^ of the total burden of hypertension still remains undiagnosed, although there is limited evidence in specific settings.[11,14]

Thus, the current study (based on WHO-STEPS approach) was conducted in a large representative adult population of North India with the following objectives:

1. estimate the prevalence of hypertension and pre-hypertension and their risk factors
2. assess the magnitude of undiagnosed cases of hypertension in the community, and
3. ascertain the blood pressure control status and associated factors among those on treatment for HTN

## Methods

### Study setting

Punjab is a state in northwest region of India bordering Pakistan and is one of the most prosperous states with a population of 2.7 million according to 2011 national census.[16] Ranked second in terms of Human Development Index among all states,[17] Punjab is called the “food basket” of India contributing nearly two thirds to the total production of food grains and a third of milk production. Their per capita income is twice that of national average. Nearly 37% of the population reside in urban areas; literacy rate is 77% and sex ratio is 893 males per 1000 males. [16,18]

### Study design and sampling

The STEPS survey was undertaken in Punjab in 2014–2015 employing a multistage stratified sampling approach using the 2011 census sampling frame. In urban areas, a three-stage procedure was followed. In the first stage, wards were selected by Probability Proportional to Size (PPS) sampling. In the second stage, one Census Enumeration Block (CEB) was randomly selected from each sampled ward. In the final stage households were selected within each CEB using systematic random sampling. The rural sample were selected in two stages: the selection of Primary Sampling Units (PSUs), which are villages by PPS at the first stage, followed by the selection of households within each PSU at second stage using systematic random sampling. Out of a total of 100 PSUs, 60 were from rural areas and 40 were CEBs from the urban locality. From each selected PSU, 54 households were selected. The ultimate sampling units were the households and one individual in the age group of 18–69 years residing in the selected household was selected using the KISH method. The details of the study methodology are described elsewhere.[19]

### Sample size

Considering the prevalence of physical inactivity as 50%[20], alpha error of 5%,design effect (1.5) and assuming a response rate of 85%, sample size was estimated to be 5400 for this study.

### Data collection instrument

A local language and pre-tested version of the WHO STEP Surveillance (STEPS) questionnaire (version 3.1) was used with minor adaptations.[21] Socio demographic and behavioural information on tobacco and alcohol use, diet, physical activity, history of chronic conditions, family history of chronic conditions, health screening, and health care costs were collected in Step 1. Physical measurements such as height, weight, blood pressure and waist circumference were collected in Step 2. Biochemical tests were conducted to measure fasting blood glucose, total cholesterol, triglycerides, HDL and LDL in Step 3 on every alternate individual. This study analyses survey data from Step 1&2 only.

#### Physical measurements (STEP 2)

Standard procedures of measurement of anthropometric variables and blood pressure mentioned below have been described in detail previously.[22] Height, weight, waist circumference were measured using standardised instruments recommended by WHO STEPS (SECA, GmbH, Hamburg, Germany). Instruments were calibrated routinely during the survey. Height and weight of participants were measured in barefoot with light clothing. Weight was measured to the nearest 10gms using an electronic scale, while height was measured to the nearest 0.1 cm using a portable stadiometer. Physical activity was assessed using the WHO Global Physical Activity Questionnaire (GPAQ).[23]

For blood pressure measurement, electronic equipment (OMRON HEM 7120, Omron Corporation, Kyoto, Japan) was used. It was validated as per the international validation protocol.[24] After rest for 5 minutes, BP was recorded in the sitting position in the right arm supported at the level of the heart. Three blood pressure measurements were taken at three minutes interval each. The final reading was recorded as the average of last two readings.

### Data analysis

Categorical variables are summarized using proportions and continuous variables using mean or median, whichever is applicable, with 95% confidence intervals. Chi-square test was used for comparison of proportions across groups and ANOVA test for comparison of means across groups. Multivariable logistic regression analysis (backward conditional method) was performed to determine the predictors of hypertension, pre-hypertension, being under treatment and control of blood pressure. Variables entered into the multivariable regression model were selected on the basis of significance (p<0.2) in the univariable analysis. Statistical analysis was done using SPSS version 16.0.

The Institute Ethics Committee of Post Graduate Institute of Medical Education and Research, Chandigarh approved the study (reference number **P-727,** dated July 21, 2014). Informed written consent was taken from all participants. The study was also ethically approved by The Union Ethics Advisory Group, Paris, France.

### Operational definitions

Cut off values recommended under WHO STEPS approach were used.[21] Current smoking was defined as those who smoked in the past 30 days and current alcohol use as those who had consumed alcohol in the last one year. Individuals who consumed less than five servings of fruits and vegetables per day were considered at risk. Sedentary activity refers to physical activity less than 600 METS per week (th minimum recommended physical activity by WHO).[23] Obesity was defined as BMI ≥27.5 kg/m^2^ which is the standard cut-off for Asian population.[25,26] Abdominal obesity was defined as a waist circumference of ≥90 cm in men and ≥80 cm in women. Hypertension was defined as systolic blood pressure (SBP) ≥140 mm of Hg, or diastolic blood pressure (DBP) ≥90 mm of Hg or the use of blood pressure-lowering medications for hypertension. Stage 1 hypertension: SBP 140–159 mm of Hg or DBP 90–99 mm of Hg; Stage 2 hypertension: SBP > = 160 mm of Hg or DBP > = 100 mm of Hg.[27] Pre-hypertension was defined as SBP lying between 120–139 mm of Hg or DBP between 80–89 mm of Hg. Isolated systolic hypertension (ISH) was defined as SBP ≥140 mm of Hg and DBP <90 mm of Hg; Isolated Diastolic Hypertension (IDH) was defined as SBP <140 mm of Hg and DBP ≥90 mm of Hg. Control of blood pressure was defined as individuals with blood pressure lower than 140/90mmHg. Individuals with fasting capillary blood glucose of ≥126 mg /dl or on medications for high blood sugar were considered to have diabetes mellitus.[28] Similarly raised intake of sodium was defined as salt intake of more than 5 grams per day.

## Results

### Socio-demographic and behavioural characteristics

Out of 5400, a total of 5127 individuals gave consent for the survey with a response rate 95%. Another 72 were removed from the analysis due to missing data thereby the effective sample size being 5055. **Table 1** shows the socio-demographic, behavioural and clinical characteristics of the respondents in the study. Majority of the respondents are females (53%), adults in the age group 25–44 years (49%), rural residents (61%) and belong to the general caste (47%). Nearly 15% were current alcohol users whereas around 6% were found to be current smokers. Around 96% of them used to have <5 servings of fruits and vegetables daily.

**Table 1:** Socio-demographic characteristic of the respondents, STEPS survey, Punjab, India, 2014–15.

Characteristics	N (%)
**Age group**	
18–24 years	804(16)
25–44 years	2491(49)
45–69 years	1760(35)
**Gender**	
Male	2373(47)
Female	2682(53)
**Residence**	
Rural	3064(61)
Urban	1991(39)
**Social group**	
SC/ST	1900(39)
Other backward caste	691(14)
General	2373(47)
**Educational status**	
Illiterate	1199(23)
Upto primary education	1249(25)
Upto secondary education	749(15)
Higher education	1858(37)
**Marital status**	
Never married	827(17)
Currently married	3818(76)
Separated/Divorced	62(1)
Widowed /cohabitating	314(6)
**Current smoking**	
Yes	317(6) )
No	4738(94)
^**a**^**Harmful alcohol use**^**¥**^	
No	902(85)
Yes	156(15)
^**b**^**Obesity**	
Yes	1286(25)
No	3769(75)
**Sedentary activity**	
Yes	4997(99)
No	58(1)
**<5 servings of fruits and vegetables daily**	
Yes	4847(96)
No	208(4)

figures represent numbers with percentages in parentheses

^**a**^one who has drank alcohol in the past 12 months.

^**b**^Obesity (Asian cut off): > = 27.5 kg/m^2^

SC: Scheduled Caste, ST: Scheduled Tribe.

### Burden of hypertension

**Fig 1:**
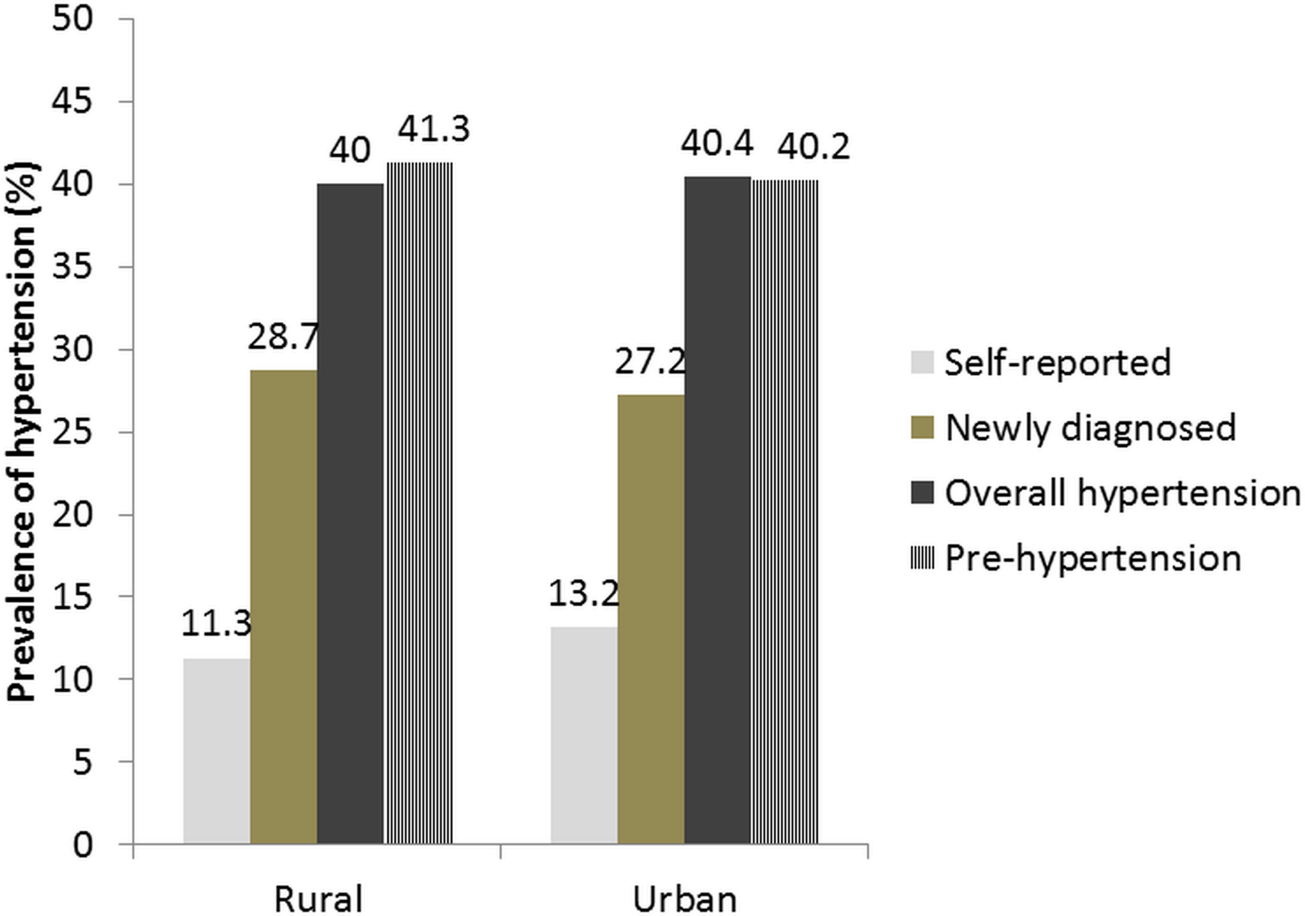
Prevalence of pre-hypertension and hypertension (self-reported and newly diagnosed) by place of residence, Punjab, India, 2014.

Overall prevalence of HTN among the study participants was found out to be 40.1% (95% CI: 38.8–41.5%) whereas the prevalences of isolated systolic hypertension, isolated diastolic hypertension and prehypertension were 6.5% (95% CI: 5.9–7.2%), 9.2% (95% CI: 8.4–10.0%) and 40.8% (39.5–42.2%) respectively. **(Table 2)** Fig 1 shows the prevalence of hypertension (self-reported and newly diagnosed) and pre-hypertension by urban and rural residence. Among all hypertensive patients (n = 2030), 1218 (60%) had stage 1 and 812 (40%) had stage 2 hypertension. The prevalence of stage 2 HTN was higher among previously diagnosed cases of HTN (304/611, 50%), compared to newly diagnosed hypertensive subjects (505/1419, 36%).

**Table 2:** Prevalence of hypertension, isolated systolic hypertension and pre-hypertension among adults (> = 18 years) by age, sex and residence in Punjab, India, 2015.

Characteristics	Hypertension	ISH	IDH	Pre-hypertension
	N (%)	N (%)	N (%)	N (%)
**Age group**				
18–24	152(18.9)	41(5.1)	46(5.7)	334(41.5)
25–44	827(33.2)	86(3.5)	277(11.1)	1091(43.8)
45–69	1051(59.7)	202(11.5)	140(7.9)	639(36.3)
**Sex**				
Male	1093(46.1)	160(6.7)	264(11.1)	1122(47.3)
Female	937(34.9)	169(6.3)	199(7.4)	942(35.1)
**Residence**				
Rural	1225(40.0)	191(6.2)	275(9.0)	1264(41.3)
Urban	805(40.4)	138(6.9)	188(9.4)	800(40.2)

ISH = Isolated Systolic Hypertension; ISH = systolic blood pressure ≥140 mm of Hg and a diastolic blood pressure <90 mm of Hg; IDH = Isolated Diastolic Hypertension; IDH = systolic blood pressure <140 mm of Hg and a diastolic blood pressure ≥90 mm of Hg; Hypertension is defined as systolic blood pressure ≥140 mm of Hg or a diastolic blood pressure ≥90 mm of Hg or already known case of HTN; Pre-hypertension = systolic blood pressure between 120–139 mm of Hg or diastolic blood pressure in the range 80–89 mm of Hg

**Table 3** shows the mean SBP and DBP in different population groups. Both the mean SBP and DBP were significantly higher in hypertensive subjects compared to normotensives (p<0.001), whereas they were not significantly different in those who were previously diagnosed and were on treatment compared to those who were newly diagnosed.

**Table 3:** Mean blood pressure in different population groups in Punjab, India, 2015.

Population sub-groups	Mean SBP (sd)	Mean DBP (sd)
Overall population	130 (20)	85 (12)
Normotensive subjects	119 (11)	78 (7)
Hypertensive subjects	147 (18)^a^	95 (11)^a^
Previously diagnosed and on treatment	144 (23)	91 (14)
Newly diagnosed hypertensives	149 (15)^b^	96 (9)^b^

Figures expressed are blood pressure values in mm of Hg; sd = standard deviation

^a^ significant difference in mean SBP and DBP in hypertensive subjects compared to normotensives

^b^ no significant difference in mean SBP and DBP in those who were newly diagnosed compared to those who were previously diagnosed and were on treatment

SBP = Systolic Blood Pressure; DBP = Diastolic Blood Pressure

### Treatment and control status of HTN

Among all persons with HTN, only 30.1% were known case of HTN or on treatment whereas the rest were newly diagnocsed or untreated. Among those already on treatment or known cases of HTN, 61% had controlled blood pressure. The proportion of undiagnosed cases of hypertension is more among males and younger individuals. **Table 4**

**Table 4:** Proportion of hypertensive patients on treatment and status of blood pressure control among those on treatment, STEPS survey, Punjab, 2014–15.

Demographic variables	Total hypertensives N	On treatment N (%)	Controlled N (%)
**Total**	N = 2030	N = 611	N = 373
**Gender**			
Male	1093	197(18.0)	100(50.8)
Female	937	414(44.2)	273(65.9)
**Age (in years)**			
18–24 years	152	15(9.9)	12(80.0)
25–44 years	827	199(24.1)	137(68.8)
45–69 years	1051	397(37.8)	224(56.4)
**Residence**			
Rural	1225	347(28.3)	203(58.5)
Urban	805	264(32.8)	170(64.4)

### Risk factors for HTN

On univariate analysis, the prevalence of HTN was found to be significantly associated with older age group (45–69 years), male gender, social group, marital status, obesity, diabetes and salt intake. Age group (45–69 years), male gender, social group, marital status, alcohol use, obesity and salt intake (> = 5 gms/day) were found to be the risk factors significantly associated with HTN in the multivariate regression model. **Table 5**

**Table 5:** Socio-economic, behavioural and clinical correlates of patients with hypertension**, STEPS survey, Punjab, India, 2014–15.

Characteristics	Total	Hypertension	p-value	Adjusted OR (95% CI)	p-value
**Age group**			**0.001**		
18–24 years	804	152(18.9)		1.0	
25–44 years	2491	827(33.2)		1.6(1.3–2.0)	**<0.001**
45–69 years	1760	1051(59.7)		4.4(3.4–5.6)	**<0.001**
**Gender**			**0.001**		
Male	2373	1093(46.1)		1.9(1.7–2.2)	**<0.001**
Female	2682	937(34.9)		1.0	
**Residence**			0.40		
Rural	3064	1225(40.0)			
Urban	1991	805(40.4)			
**Social group**			**0.001**		
SC/ST	1900	703(37.0)		1.0	
Other backward caste	691	267(38.6)		1.1(1.0–1.3)	0.1
General	2373	1026(43.2)		1.4(1.2–1.6)	**<0.001**
**Educational status**			**0.01**		
Illiterate	1199	533(44.5)		1.0	
Upto primary education	1249	542(43.4)		1.0(0.9–1.2)	0.2
Upto secondary education	749	283(37.8)		1.0(0.8–1.2)	0.3
Higher education	1858	672(36.2)		0.9(0.8–1.1)	0.2
**Marital status**			**0.001**		
Never married	827	179(21.6)		1.0	
Currently married	3818	1625(42.6)		1.5(1.2–1.9)	**<0.001**
Separated/Divorced/Widowed	376	211(56.1)		2.5(1.8–3.5)	**<0.001**
**Current smoking**			0.40		
Yes	317	128(40.4)			
No	4738	1902(40.1)			
**Current alcohol use**			**0.09**		
Yes	902	491(54.4)		1.3(1.1–1.5)	**0.01**
No	156	69(44.2)		1.0	
^**a**^**Diabetes**			**0.001**		
Yes	193	129(66.8)		**1.8 (1.6–2.0)**	**<0.001**
No	2279	834(36.6)		**1.0**	
**> = 5 servings of fruits and vegetables daily**			0.3		
Yes	208	79(38.0)			
No	4847	1951(40.3)			
^**b**^**Obesity**			**0.001**		
Yes	1286	732(56.9)		2.4(2.0–2.8)	**<0.001**
No	3769	1298(34.4)		1.0	
**Family history of high blood pressure**			0.2		
Yes	1682	695(41.3)			
No	3312	1322(39.9)			
**Sedentary activity**			0.3		
Yes	4997	31(53.4)			
No	58	1999(40.0)			
**Salt intake**			**0.01**		
<5 gms/day	3806	1256(33.0)		1.0	
> = 5 gms/day	1249	508(40.7)		1.2(1.0–1.4)	**0.02**

Backward conditional multivariable logistic regression performed; Values are presented as Odds ratio OR (95% Confidence Interval, CI), p value

** SBP≥140 and/or DBP≥90 or currently on medication

SC/ST stands for Scheduled Caste/Scheduled Tribe

^a^Individuals with fasting capillary blood glucose of ≥126 mg /dl or on medications for high blood sugar were considered to have diabetes

^b^ Obesity is defined as BMI≥27.5 kg/m2

**Table 6** shows the factors associated with hypertension by the type of residence (rural and urban) and in different age groups (18–24 years and 45–69 years). Age >24 years and male gender were significantly associated with hypertension in both rural and urban residences. Being a current alcoholic, diabetic and obese were associated with HTN in urban areas, whereas association between salt intake (> = 5 gms/day) and HTN was found only in rural areas. Among respondents aged 18–24 years, male gender, urban residence, illiteracy and obesity were found to be the factors associated with HTN.

**Table 6:** Socio-economic, behavioural and clinical correlates of patients with hypertension by place of residence and age group, STEPS survey, Punjab, India, 2014–15.

Characteristics	Hypertension Hypertension			
	Rural residence	Urban	18–24 years	45–69 years
**Age group**				
25–44 years	1.5 (1.1–2.2)*	2.7(1.9–4.0)**		
45–69 years	3.6(2.3–5.4)**	10.5(7.2–15.6)**		
**Male gender**	2.0 (1.6–2.6)**	1.6 (1.3–1.9)**	3.5 (2.0–6.0)**	1.8(1.3–2.5)**
**Urban residence**			2.0 (1.1–3.7)*	1.4 (1.0–1.9)*
**General caste**	-	-	-	-
**Illiteracy**	-	-	2.0 (1.1–4.0)*	-
**Separated/Divorced/**	-	-	-	3.9(1.1–12.3)*
**Widowed**				
**Current smokers ^**a**^**	-	-	-	-
**Current alcoholics ^**b**^**	-	1.4(1.1–1.6)*	-	-
**Diabetic ^**c**^**	-	1.6 (1.2–2.0)*	-	-
**Obesity ^**d**^**	-	2.1 (1.6–2.8)*	6.0(2.5–14.0)**	2.1 (1.4–3.2)*
**Salt intake**	1.2(1.0–1.4)*	-	-	-
**(> = 5 gms/day)**				
**Family history of HTN**	-	-	-	1.5 (1.1–2.1)*
**>5 servings of fruits**	-	-	-	-
**and vegetables daily**				

Backward conditional multivariable logistic regression performed; Values are presented as Odds ratio OR (95% Confidence Interval, CI)

*p value<0.05

** p value<0.001; HTN = Hypertension

^a^ current smoker defined as smoking in the last 30 days

^b^ current alcoholic defined as one who has drank alcohol in the last 12 months

^c^Individuals with fasting capillary blood glucose of ≥126 mg /dl or on medications for high blood sugar were considered to have diabetes

^d^ Obesity is defined as BMI≥27.5 kg/m^2^ Hypertension is defined as systolic blood pressure ≥140 mm of Hg or a diastolic blood pressure ≥90 mm of Hg or already known case of HTN

### Factors associated with pre-hypertension, being on treatment and control of blood pressure

The factors associated with pre-hypertension are age>25 years, male gender, obesity, diabetes, current smoking and salt intake > = 5gms/day. Among those who were previously diagnosed and already on treatment, males (0.4, 0.2–0.7), obese (0.6, 0.4–0.8) and those with sedentary activity (0.5, 0.2–0.9) are less likely to have controlled blood pressure. **Table 7.**

**Table 7:** Socio-economic, behavioural and clinical correlates of pre-hypertension, being previously diagnosed with hypertension and on treatment among all hypertensives and having controlled blood pressure among those who are already on treatment, STEPS survey, Punjab, India, 2014–15.

Characteristics	Prehypertension	Already on treatment	Controlled blood pressure
**Age group**			
25–44 years	1.5 (1.1–2.1)*	-	-
45–69 years	1.9 (1.2–2.9)*	2.1 (1.6–3.8)*	-
**Male gender**	3.7 (2.8–4.9)**	0.3 (0.2–0.5)**	0.4 (0.2–0.7)*
**Urban residence**	-	-	-
**General caste**	-	-	-
**Illiteracy**	-	-	-
**Separated/Divorced/**	-	-	-
**Widowed**			
**Current smokers** ^**a**^	1.8 (1.2–3.0)*	-	-
**Current alcoholics** ^**b**^	-	-	-
**Diabetic** ^**c**^	2.1 (1.2–3.9)*	1.7(1.2–2.6)*	0.6 (0.3–0.8)*
**Obesity** ^**d**^	1.4 (0.97–2.1)	1.5(1.1–2.0)*	0.6 (0.4–0.8)*
**Salt intake**	1.2(1.0–1.4)*	-	-
**(> = 5 gms/day)**			
**Family history of HTN**	-	1.4(1.1–1.8)*	-
**>5 servings of fruits**	-	3.7(1.6–6.4)*	-
**and vegetables daily**			
**Sedentary activity**		-	0.5 (0.2–0.9)*

Backward conditional multivariable logistic regression performed; Values are presented as Odds ratio OR (95% Confidence Interval, CI)

*p value<0.05

** p value<0.001; HTN = Hypertension

^a^ current smoker defined as smoking in the last 30 days

^b^ current alcoholic defined as one who has drank alcohol in the last 12 months

^c^ Individuals with fasting capillary blood glucose of ≥126 mg /dl or on medications for high blood sugar were considered to have diabetes

^d^ Obesity is defined as BMI≥27.5 kg/m^2^; Pre-hypertension = systolic blood pressure between 120–139 mm of Hg or diastolic blood pressure in the range 80–89 mm of Hg

## Discussion

The key findings of the present study are: 1) Around 40% of the general adult population have raised blood pressure or are known case of HTN; 2) ISH and IDH were found to be in 6.5% and 9.2% respectively; prehypertension was found in 40.8% of the population 3) Factors strongly associated with hypertension include older age group (45–69 years), male gender, social group, marital status, alcohol use, obesity and salt intake (> = 5 gms/day) 4) a large burden of undiagnosed cases of HTN exists among the adult population, a significant proportion of whom have uncontrolled blood pressure levels. 5) Males, obese patients, those with sedentary lifestyle and patients with diabetes were more likely to have uncontrolled blood pressure. It is highly likely that the high burden of pre-hypertension and hypertension is going to be the key driver of the epidemic of cardiovascular disease in India.

Several studies have reported the prevalence of HTN in the range of 30–47% similar to the figures presented in this study.[4,8–10,12] The Prospective Urban Rural Epidemiology (PURE) study, which recruited 26 861 individuals aged 35 to 70 years between 2003–2009 in India reported the prevalence of HTN to be 30.7%.[29] A systematic review found the overall prevalence of hypertension in India to be 29.8%.[13] Another large nationwide study (ICMR-INDIAB study) by Bhansali et al. revealed hypertension among 26.3% of the population.[14] Alarmingly high prevalence (60%) of HTN among the elderly age group found in this study is supported by other studies in similar settings.[30]

Another worrisome finding is the high prevalence of pre-hypertension. Slightly higher figures were reported in previous studies in India among the urban adult population in India.[31,32] Persons with pre-hypertension have a greater risk of developing hypertension and are also associated with increased risk of major cardiovascular events.[33,34] The excess risk associated with prehypertension and progression to HTN can be prevented by reducing BP through non-pharmacologic (dietary modification, weight loss, reduced sodium intake, regular physical activity and moderation of alcohol intake) and pharmacologic interventions (if non-pharmacologic intervention fails or in patients with certain comorbidities) as recommended by JNC-8.[27] Management of pre-hypertensives, like other non-communicable diseases, requires a cohort-wise registration and follow-up mechanism to monitor and improve the delivery of the intervention.[35] This requires considerable strengthening of the public health system. The JNC 8 calls for routine blood pressure measurement at least once every 2 years for adults with pre-hypertension.[27] Surveillance of this pre-HTN population particularly among those >40 years of age for early detection of HTN will be essential.

High BMI was independently associated with HTN especially in urban areas which is similar to the results in most other studies.[5–7,9,11,14,36–38] Similarly, age was also significantly associated with HTN with very high prevalence in the elderly age group.[4–9,11,14,29,30,36–39]

Salt intake was found to be associated with HTN as reported in previous studies.[5,7,12,14,37] Excessive sodium intake (>2g/d) is an important cause of high blood pressure and estimated to cause 1.65 million cardiovascular related deaths each year.[40] A Cochrane systematic review has demonstrated significant fall in blood pressure following modest reduction in salt intake in both hypertensive and normotensive individuals.[41] The World Health Organization (WHO) has recommended salt reduction as a ‘best buy’, recognising it as one of the most cost effective and feasible approaches to prevent non-communicable diseases (NCDs).[42] A systematic review on salt reduction initiatives around the world showed that 75 countries have a national salt reduction strategy, although activity remains limited in low- and middle-income regions. The majority of programs are multifaceted and include industry engagement to reformulate products, establishment of sodium content targets for foods, consumer education, front-of-pack labelling schemes, taxation on high-salt foods and interventions in public institutions.[43] It’s high time India formulated a multifaceted salt reduction strategy which would facilitate India to realise the global target of reduction in mean population salt intake by 30% and thus reduce associated premature morbidity and mortality.[44]

The present study showed high prevalence of HTN among males similar to other large cross-sectional studies,[8,14] although there is conflicting evidence in the literature which show no gender difference.[4–6,9] The present study reported no urban rural difference in the prevalence of HTN. In contrast, earlier studies have reported significant urban-rural differences[13,14] which probably points towards the equalisation of the urban rural divide in recent times even in the context of other non-communicable diseases and their risk factors.[45]

The analyses show that nearly 70% of individuals with HTN were previously undiagnosed or untreated. Similar figures are also reported by few studies from India which put the proportion of undiagnosed cases in the community in the range of 60–80%, thereby indicating the need for aggressive screening programs.[11,14,30,36,38] A large cross-sectional study across 17 countries (Prospective Urban Rural Epidemiology (PURE) study) by Chow et al. reported that only 46.5% were aware of their diagnosis.[29] Another five-city urban study in India (Kolkata, Nagpur, Mumbai, Thiruvananthapuram and Moradabad) revealed that only a quarter of hypertensive patients were aware of their diagnosis.[37] The pool of undiagnosed cases of HTN left untreated is more prone to complications and morbidities such as CHD, cardiac failure, cerebral stroke, damage to blood vessels etc. Hence, it is necessary to identify and offer early therapy to these individuals and ensure regular follow up. However, the study showed that around 61% of the patients on treatment have controlled blood pressure which is higher than other studies in India which have reported control status in around one-third of them. [10,29,36,46] On the other hand, Moser et al. reported controlled blood pressure in nearly two-thirds of the hypertensive patients on treatment, more among women. [38] Further studies are required to understand the patient level, community level and health system level factors associated with control of blood pressure.

There were important differences between the sexes. The higher overall rates of diagnosis and blood pressure control among women as seen in other studies[29,38] probably result from contact with health services around childbearing and also consistent with a large body of evidence stating better health seeking behaviour among women.[38,47] The failure to detect hypertension in younger individuals, and the poor diagnosis and control among men, is of particular importance, suggesting a need for improved focus on specific population groups.

This study showed that patients with uncontrolled BP were more frequently male, obese patients, with sedentary lifestyle and patients with diabetes which is well supported in the literature from different settings.[48–50] The large burden of undiagnosed HTN and poor control (a measure of inadequate treatment) is a concern. The low rates of detection and control may be because few individuals get their blood pressure checked through routine health assessment due to poor access or costs in accessing health care. Also, there is no regular screening program or a mechanism of regular timely follow-up for patients with HTN and other non-communicable diseases in India.

### Strengths and limitations

The strengths of the study are that it is population-based, employed a large multistage stratified sample representative of the general adult population with a high response rate and followed a robust methodology (WHO-STEPS approach). The study also adhered to STROBE guidelines for reporting the results.[51] The present study had few limitations. This, being a cross-sectional study, causal pathways underlying the reported associations could not be ascertained. Also, information on blood pressure lowering drug therapy could not be collected in this study.

## Conclusion

The study reported alarmingly high prevalence of hypertension among the adult population in a representative North Indian population, calling for an immediate attention. The study also highlights a significant burden of undiagnosed or untreated cases of HTN in the community. This indicates the need for systematic screening and awareness program to identify the undiagnosed cases in the community and offer early treatment and regular follow up in order to prevent complications and premature mortality.

## Supporting information

S1 File Dataset. (XLSX)
